# A review of the dragon millipede genus *Desmoxytes* Chamberlin, 1923 in China, with descriptions of four new species (Diplopoda, Polydesmida, Paradoxosomatidae)

**DOI:** 10.3897/zookeys.448.8081

**Published:** 2014-10-20

**Authors:** Weixin Liu, Sergei I. Golovatch, Mingyi Tian

**Affiliations:** 1 College of Natural Resources and Environment, South China Agricultural University, 483 Wushanlu, Guangzhou, 510640, China; 2 Institute for Problems of Ecology and Evolution, Russian Academy of Science, Leninsky pr. 33, Moscow 119071, Russia

**Keywords:** Diplopod, *Desmoxytes*, new species, cave, troglobite, key, China

## Abstract

Four new species of *Desmoxytes* are described from southern China: *Desmoxytes lingulata* **sp. n.**, *Desmoxytes parvula* **sp. n.**, and *Desmoxytes nodulosa* **sp. n.**, from Guangxi Zhuang Autonomous Region, and *Desmoxytes getuhensis* **sp. n.** from Guizhou Province. In addition, new records of *Desmoxytes scutigeroides* Golovatch, Geoffroy & Mauriès, 2010 and *Desmoxytes scolopendroides* Golovatch, Geoffroy & Mauriès, 2010 are provided, with a modified key to *Desmoxytes* species currently known to occur in China. Two of the new species, *Desmoxytes nodulosa* **sp. n.** and *Desmoxytes getuhensis* **sp. n.**, seem to be troglobites.

## Introduction

*Desmoxytes* Chamberlin, 1923 is a large, common, rather well defined, southeast Asian genus of the basically oriental millipede tribe Orthomorphini, subfamily Paradoxosomatinae, family Paradoxosomatidae (Golovatch et al. 2012). The genus is one of the very few among paradoxosomatid millipedes which not only harbours troglobitic species, but also bears its own vernacular name, the “dragon millipedes”, labeled so to emphasize the unusually prominent, wing-, spine- or antler-shaped paraterga. At the moment, *Desmoxytes* is represented by 29 species, usually aposematic, brightly coloured and surface-active, ranging from southern China in the north, through Indochina, down to approximately the middle of Malay Peninsula within both Thailand and Malaysia in the south (Golovatch et al. 2012). Only one species, *Desmoxytes planata* Pocock, 1895, has attained a vast, nearly pantropical distribution through human agency (Nguyen and Sierwald 2013).

At present, China supports 10 species of *Desmoxytes*, including 7 presumed troglobites. Unlike the epigean congeners usually demonstrating bright live colorations, the cavernicolous *Desmoxytes* are typically poorly pigmented and appear to be confined to caves in southern China while the genus is the sole among oriental Paradoxosomatidae to contain troglobites (Golovatch et al. 2010, 2012). The following species of *Desmoxytes* have hitherto been known to occur in continental China:

*Desmoxytes cornuta* Zhang & Li, 1982, from Guangxi, Guilin, Yangshuo (Zhang and Li 1982).

*Desmoxytes draco* Cook & Loomis, 1924, from Jiangxi, Jiujiang, Lushan Mountains (Cook and Loomis 1924).

*Desmoxytes eupterygota* Golovatch, Li, Liu & Geoffroy, 2012, from two caves in Hunan, Chenzhou, Linwu (Golovatch et al. 2012).

*Desmoxytes longispina* Loksa, 1960, from a cave in Guangxi (no exact locality is known) (Loksa 1960; Golovatch et al. 2010, 2012).

*Desmoxytes lui* Golovatch, Li, Liu & Geoffroy, 2012, from a cave in Guangxi, Yongfu (Golovatch et al. 2012).

*Desmoxytes minutubercula* Zhang, 1986, from Guangxi, Tianlin (Zhang 1986).

*Desmoxytes planata* Pocock, 1895, from a cave in Yunnan, Luxi, but basically nearly pantropical (Pocock 1895; Zhang 1986).

*Desmoxytes scolopendroides* Golovatch, Geoffroy & Mauriès, 2010, from a cave in Guangxi, Huanjiang (Golovatch et al. 2010).

*Desmoxytes scutigeroides* Golovatch, Geoffroy & Mauriès, 2010, from several caves in Guangxi, Huanjiang (Golovatch et al. 2010).

*Desmoxytes spinissima* Golovatch, Li, Liu & Geoffroy, 2012, from a cave in Guangxi, Fuchuan. (Golovatch et al. 2012).

The present paper describes a further four new species of *Desmoxytes*, two of which seem to be troglobites, as well as provides new records of two known presumed troglobitic congeners.

## Material and methods

The holotypes and a number of paratypes are deposited in the zoological collection of the South China Agricultural University, Guangzhou, China (SCAU), with some material also to be housed in the Institute of Zoology, Chinese Academy of Sciences, Beijing, China (IZAS), and Zoological Museum, State University of Moscow, Russia (ZMUM). The methods and terminology used are after Golovatch et al. (2012).

## Taxonomic part

### Desmoxytes lingulata sp. n.

Taxon classificationAnimaliaPolydesmidaParadoxosomatidae

http://zoobank.org/2C15FE60-AD85-4A8E-83A8-1577D277735E

Figs 1 , 2 , 3

#### Holotype.

♂ (SCAU), China, Guangxi, Guilin City, Pingle County, Ertang Town, Chaotianyan, 24°37.075'N, 110°45.501'E, 257 m, 29.IV.2013, leg. Tian Mingyi, Liu Weixin, Sun Feifei & Yin Haomin.

#### Paratypes.

3 ♂ (SCAU), same locality and collecting data as of the holotype.

#### Name.

To emphasize a peculiar, paramedian, linguiform, sternal process between ♂ coxae 5.

#### Diagnosis.

Differs from congeners in the paraterga being antler-shaped, the humped ♂ femur 6, combined with small, setose tubercles between ♂ coxae 3 and a peculiar sternal process between ♂ coxae 5, as well as the shout and curved gonopod femorite and a condensed solenophore.

#### Description.

**Figure 1.**
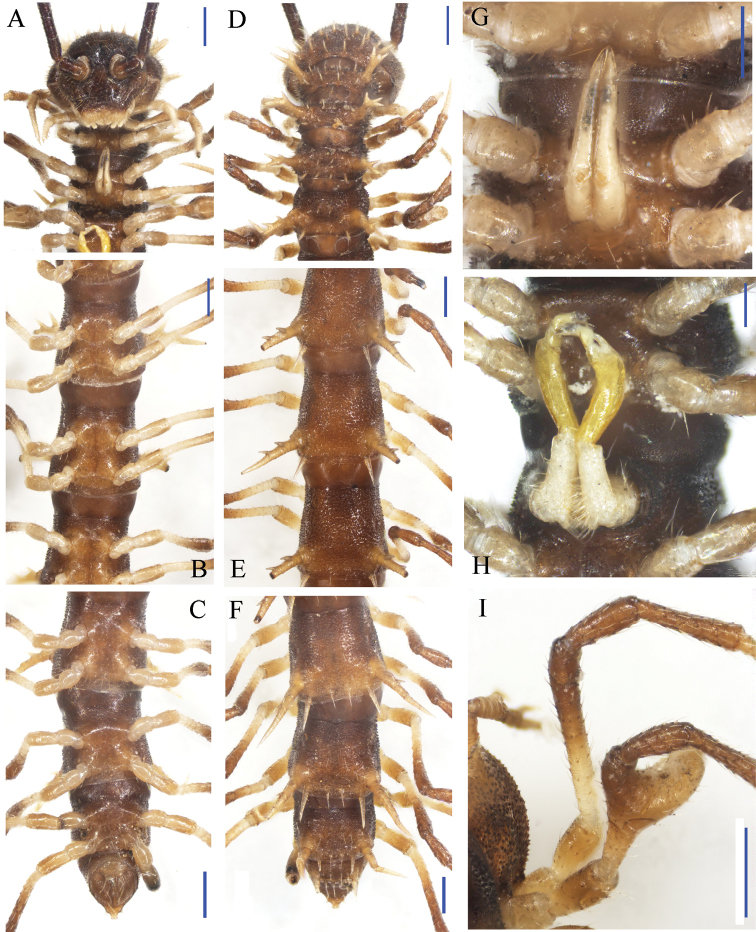
*Desmoxytes lingulata* sp. n., ♂ paratype from Ertang Twon, Chaotianyan. **A, D** anterior part of body, ventral and dorsal views, respectively **B, E** midbody segments, ventral and dorsal views, respectively **C, F** posterior part of body, ventral and dorsal views, respectively **G** sternal process in the middle of ♂ sternum 5 *in situ*, ventral view **H** gonopods *in situ*, ventral view **I** femur 6, lateral view. Scale bars: **A–F, I** = 0.5 mm; **G, H** = 0.2 mm.

Length ca 18.0–18.5 mm (♂), width of pro- and metaterga together with paraterga 0.8–1.0 and 1.8–2.0 mm (♂), respectively. Holotype 18.0 mm long, 0.8 and 2.0 mm wide on midbody pro- and metazonae, respectively. Head broadest, 1.2–1.4 mm (♂) (Fig. 1D). Coloration of material rather uniformly dark brownish (Fig. 1). Antennomeres 5 and 6, paraterga, posterior parts of metaterga, and sterna brownish to yellow brownish; apex of antennomere 7 pallid; a few basal podomeres yellowish (Fig. 1). Head densely setose, epicranial suture distinct (Fig. 1A). Antennae rather long and slender, reaching back until segment 7 or 8 (♂) when stretched dorsally, antennomeres 5 and 6 each with a compact apicodorsal group of bacilliform sensilla.

**Figure 2.**
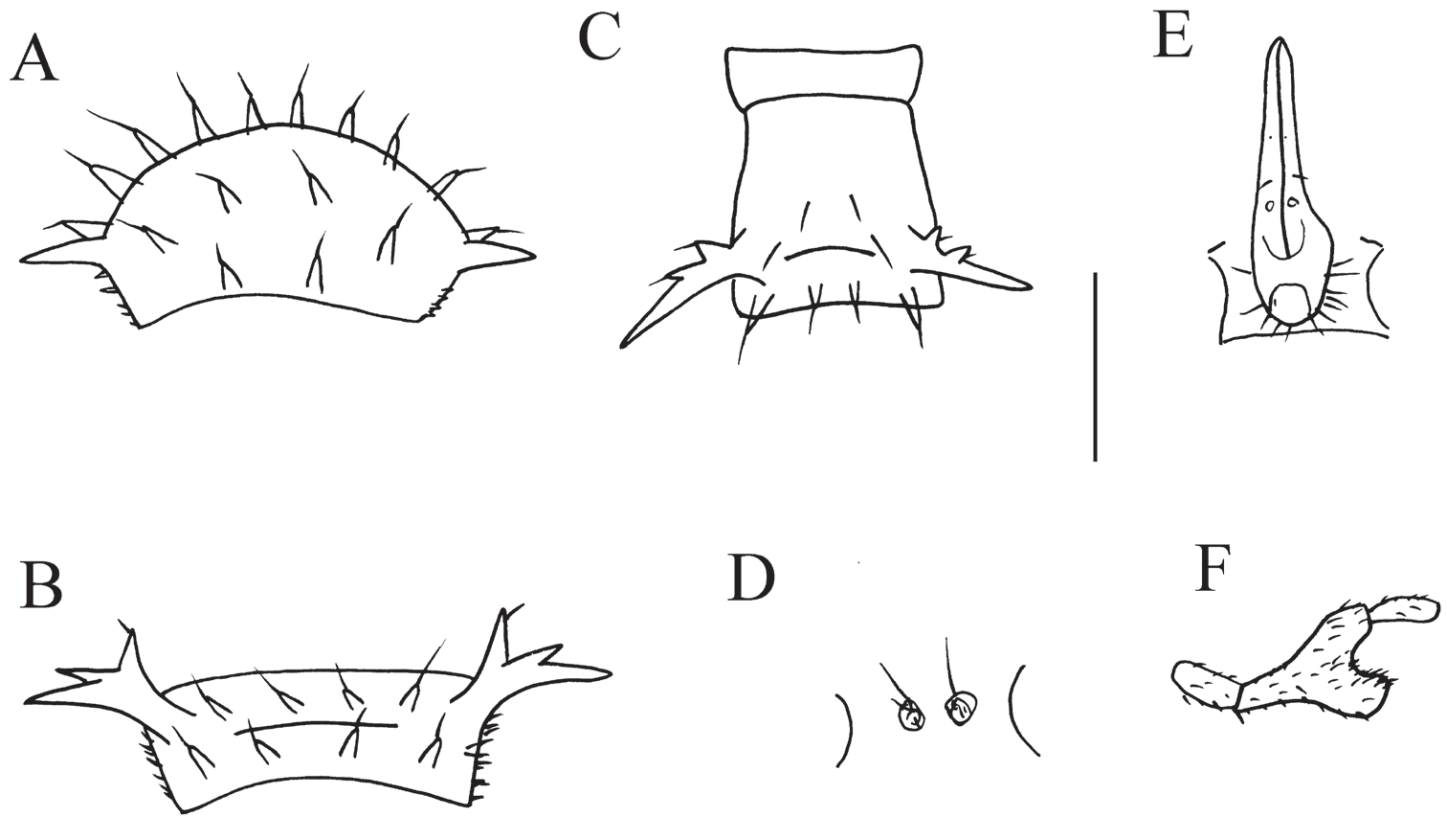
*Desmoxytes lingulata* sp. n., ♂ paratype from Ertang Twon, Chaotianyan. **A** Collum **B** metatergum 2, dorsal view **C** segment 10, dorsal view **D** sternal cones between coxae 3, ventral view **E** sternal processes between coxae 5, ventral view **F** femur 6, front view. Scale bar: **A–B, D–E** = 0.5 mm; **C, F** = 1.0 mm.

Prozonae very delicately microalveolate; surface below paraterga 2–4 rather shagreened and microspinulate (Fig. 2B), surface below following paraterga and metaterga finely microgranulate and moderately setose (Fig. 1D, E). Collum with three transverse rows of large, setigerous spines: 4+4 anterior, 2+2 intermediate, 1+1 posterior; paraterga stout and spiniform, directed dorsolaterad, with a setigerous spine anteriorly at base (Figs 1D, 2A). Metaterga 2–4 with 2+2 and 2+2 large setigerous spines arranged in two transverse rows (Figs 1D, 2B); metaterga 5–18 with three transverse rows of setigerous spines: 1+1 anterior; 1+1 intermediate, located at base of paraterga; 2+2 posterior, lateral spines of posterior rows much larger than the others in metaterga 2–18 (Figs 1E, 2C); metatergum 19 with 2+2(3) anterior and 2+2(3) posterior rows of setigerous spines of same size (Fig. 1F). Paraterga antler-shaped, very strongly developed, ca 0.8–1.0 times as long as body height. Paraterga 2–4 subvertical (Fig. 2B); following paraterga 5–18 rather long, evidently 2- or 3-dentate laterally, near tip of each denticle with a seta, directed dorsolaterally and ending up clearly above dorsum (Figs 1E, 2C); paraterga 19 short spines directed caudad (Fig. 1F). Ozopores rather inconspicuous. Transverse sulcus visible on metaterga 2–18. Pleurosternal carinae very evident on ♂ segments 2 and 3, obscure on the rest. Epiproct with 2+2 setigerous tubercles on lateral sides, and 1+1 paramedian ones near midway dorsally, tip subtruncate, lateral pre-apical papillae very distinct, tuberculiform. Hypoproct subtrapeziform, caudal margin very slightly concave, setigerous cones at caudal edge very small, widely separated (Fig. 1C, F). Axial line missing.

Sterna sparsely setose, cross-impressions faint (Fig. 1B). A paramedian pair of entirely separated, very small, setose tubercles between ♂ coxae 3 (Fig. 2D). A peculiar, paramedian, linguiform sternal process between ♂ coxae 5 (Figs 1A, G, 2E). Legs 1 short, following ones increasingly longer and slenderer towards telson, ca 3.5–4.0 (♂) times longer than body height. ♂ femur 6 with a very strong, mesal, distoventrally densely pilose apophysis in distal half (Figs 1I, 2F).

**Figure 3.**
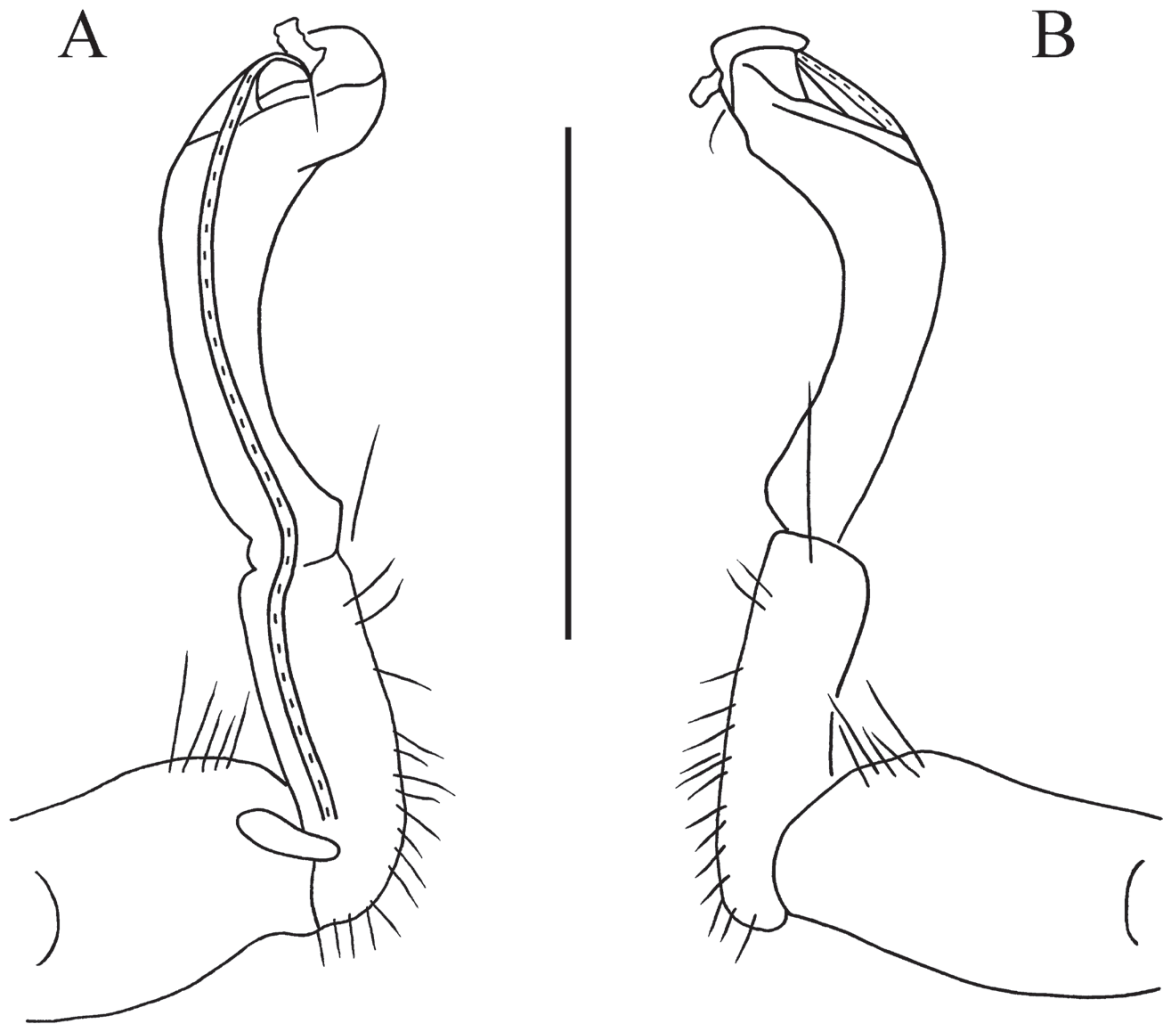
*Desmoxytes lingulata* sp. n., ♂ paratype from Ertang Twon, Chaotianyan. **A, B** left gonopod, mesal and lateral views, respectively. Scale bar: 0.5 mm.

Gonopods (Figs 1H, 3A, B) subfalcate. Coxite subcylindrical, poorly setose distodorsally, about 1/3 as long as telopodite. Prefemoral portion rather long, about as long as acropodite, densely setose. Femorite short, curved dorsad, with seminal groove running entirely on mesal side, apically with a strongly condensed solenophore. Solenomere short, flagelliform, folded apically, rather faintly separated at base from solenophore.

#### Remarks.

This species seems to be especially similar to *Desmoxytes cornuta* Zhang & Li, 1982, from Guangxi, Guilin, Yangshuo. Obvious differences lie in a peculiar linguiform sternal process between ♂ coxae 5, combined with the stout, curved gonopod femorite and a condensed solenophore in *Desmoxytes lingulata* sp. n., as opposed to an elongated and suberect one in *Desmoxytes cornuta* (cf. Zhang and Li 1982).

### Desmoxytes parvula sp. n.

Taxon classificationAnimaliaPolydesmidaParadoxosomatidae

http://zoobank.org/0E3F9DD5-1FFE-45BB-B896-631C999232F4

Figs 4 , 5 , 6

#### Holotype.

♂ (SCAU), China, Guangxi, Hechi City, Du’an County, Xia’ao Town, cave I, 24°15.144'N, 107°56.272'E, 347 m, 2.V.2013, leg. Tian Mingyi, Liu Weixin, Sun Feifei & Yin Haomin.

#### Paratype.

1 ♀ (SCAU), same locality and collecting data as of the holotype.

#### Name.

To emphasize the small size of this species.

#### Diagnosis.

Differs from congeners in the combination of spiniform paraterga, a paramedian pair of subtrapzoidal processes between ♂ coxae 4, the humped ♂ femur 6, and certain details of gonopod structure.

#### Description.

**Figure 4.**
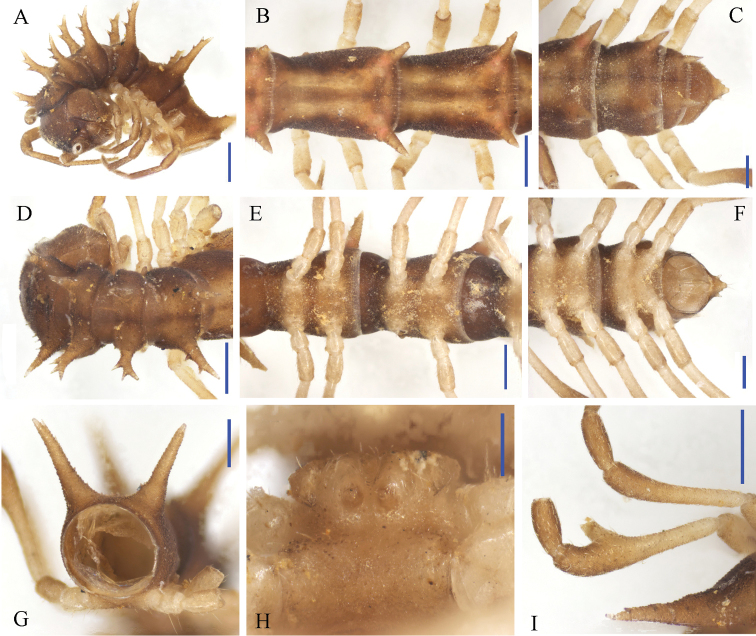
*Desmoxytes parvula* sp. n., ♂ holotype from Xia’ao Town, cave I. **A, D** anterior part of body, lateral and dorsal views, respectively **B, E** midbody segments, dorsal and ventral views, respectively **C, F** posterior part of body, dorsal and ventral views, respectively **G** cross-section of a midbody segment, frontal view **H** sternal process between coxae 4 *in situ*, ventral view **I** femur 6, lateral view. Scale bars: **A–G, I** = 0.5 mm; **H** = 0.2 mm.

Length ca 18 (♂) or 19 mm (♀), width of pro- and metaterga together with paraterga 0.8 and 1.2 (♂), or 1.0 and 1.4 mm (♀), respectively. Head broadest, 1.3 mm (♂) or 1.5 mm (♀) wide. Coloration of material rather uniformly brownish, antennae and lateral body parts dark brown, venter and a few basal podomeres yellowish, basal parts paraterga pink (Fig. 4). Head densely setose, epicranial suture distinct. Antennae long and slender, reaching back to segment 6 (♂) or 4 (♀) when stretched dorsally, antennomeres 5 and 6 each with a compact apicodorsal group of bacilliform sensilla.

**Figure 5.**
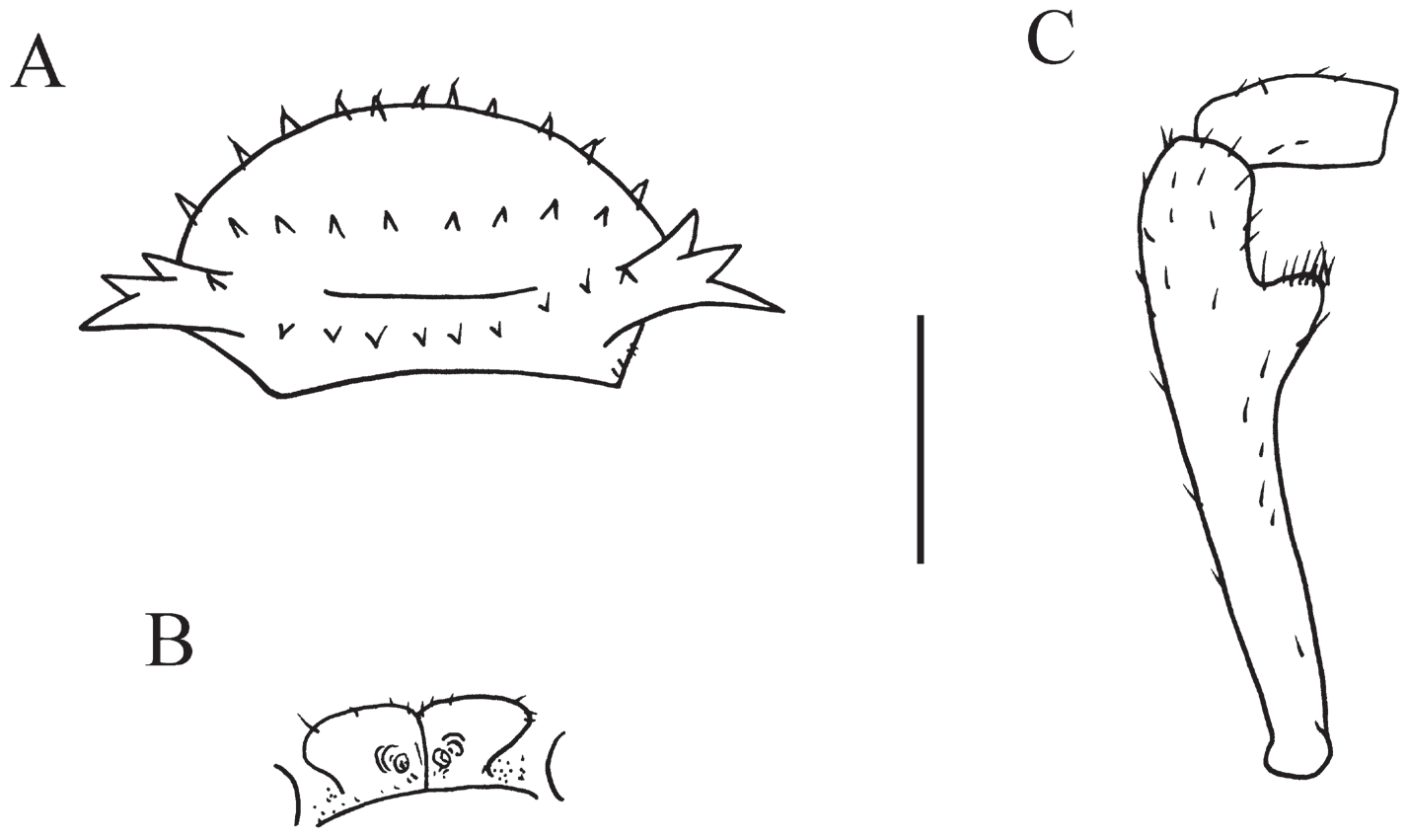
*Desmoxytes parvula* sp. n., ♂ holotype from Xia’ao Town, cave I. **A** Collum **B** sternal process between coxae 4, ventral view **C** femur 6, lateral view. Scale bar: 0.5 mm.

Pro- and metazonae very delicately microalveolate, metaterga finely shagreened and transversely rugulose, surface below paraterga finely shagreened (Fig. 4A–F). Collum with three transverse rows of rather evident spines: 5(6)+5(6) anterior, 4+4 intermediate and 4(5)+4(5) posterior, setae often visible, but sometimes obliterated (Fig. 5A); paraterga spiniform, each with 2 denticles laterally, a spine anteriorly at base (Figs 4A, 5A). Metaterga 2–4 with three transverse rows of setigerous tubercles: 4+4 anterior, 4+4 inermediate, 5+5 posterior. Starting from metatergum 5, anterior row gradually showing 1–2 additional tubercles so that following metaterga with transverse rows of 4–6 irregular tuberculations varying in number, but posterior two rows usually regular, each with (3–5)+(3–5) and (5–8)+(5–8) tuberculations (Fig. 4B). Metatergum 19 with five rather regular rows of tuberculations. Paraterga spiniform, each with 2–3 denticles (Fig. 4A–D). ♂ paraterga 2–9 subvertical, following paraterga directed dorsolaterally (Fig. 4A–B, G), but ♀ paraterga mostly low and short; paraterga 19 directed caudad (Fig. 4C). Ozopores inconspicuous. Transverse sulcus visible on coullum and metaterga 2–18 (Figs 4B–C, 5A). Pleurosternal carinae poorly developed on segments 2 and 3 both in ♂ and ♀, absent on the rest (Fig. 4D). Epiproct (Fig. 4C) simple, dorsal subapical and, especially, lateral pre-apical papillae very distinct, tuberculiform. Hypoproct (Fig. 4F) subtrapeziform, caudal margin very slightly concave, setigerous cones at caudal edge very small, widely separated. Axial line present.

Sterna moderately setose, cross-impressions very weak (Fig. 4E). A paramedian pair of subtrapzoidal processes between ♂ coxae 4 (Figs 4H, 5B). Legs 1 short, following ones increasingly longer and slenderer towards telson, ca 2.5 (♂) or 2.0 (♀) times longer than body height. ♂ femur 6 with a very evident, digitiform, distoventral apophysis in distal 1/3 (Figs 4I, 5C).

**Figure 6.**
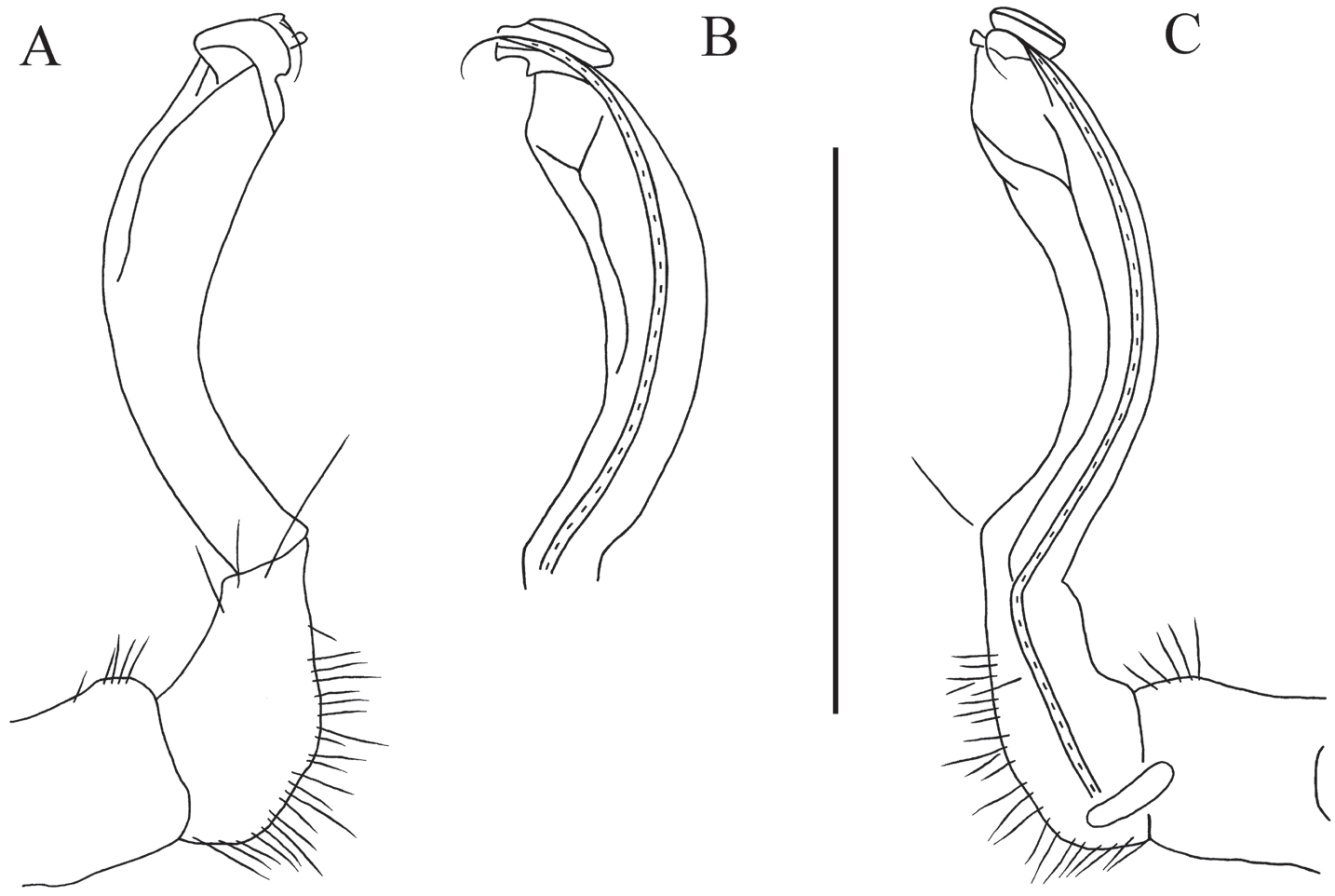
*Desmoxytes parvula* sp. n., ♂ holotype from Xia’ao Town, cave I. **A–C** right gonopod, lateral, dorsal and mesal views, respectively. Scale bar: 0.5 mm.

Gonopods (Fig. 6A–C) simple, strongly elongated. Coxite rather short, subcylindrical, poorly setose distodorsally, about 1/3 as long as telopodite. Prefemoral portion about half as long as acropodite, densely setose. Femorite rather long, strongly curved dorsad, slightly enlarged distally, with seminal groove running entirely on the mesal side. Postfemoral part strongly condensed; solenomere short, flagelliform, sheathed by a similarly short solenophore.

#### Remarks.

Even though this species has been taken from a cave, it hardly represents a true cavernicole as it is rather strongly pigmented and shows short antennae and legs.

### Desmoxytes nodulosa sp. n.

Taxon classificationAnimaliaPolydesmidaParadoxosomatidae

http://zoobank.org/D3F59C0C-A666-459B-9B51-542DB05A2AB4

Figs 7 , 8 , 9

#### Holotype.

♂ (SCAU), China, Guangxi, Hechi City, Du’an County, Xia’ao Town, near Xia’ao Middle School, cave II, 24°17.987'N, 107°57.146'E, 317 m, 3.V.2013, leg. Tian Mingyi, Liu Weixin, Sun Feifei & Yin Haomin.

#### Paratypes.

3 ♂, 4 ♀ (SCAU), 1 ♂, 1 ♀ (IZAS), 1 ♂, 1 ♀ (ZMUM), same locality, and collecting data as of the holotype. 1 ♀ (SCAU), same county, Yong’an Town, Yong’an Village, cave I, 24°14.659'N, 108°03.032'E, 287 m; 1 ♀ (SCAU), same town, Anju Village, cave Suidao Dong, 24°13.340'N, 108°05.694'E, 311 m, 3.V.2013, leg. Tian Mingyi, Liu Weixin, Sun Feifei & Yin Haomin; 1 ♂, 2 ♀ (SCAU), same county, Longwan Town, Qunle Village, cave I, 23°56.021'N, 108°10.962'E, 459 m, 27.VI.2013, leg. Tian Mingyi, Lin Wei, Liu Weixin, Yin Haomin & Huang Sunbin.

#### Name.

To emphasize the humped ♂ femora 5–7.

#### Diagnosis.

Differs from congeners in most of the paraterga being wing-shaped, combined with the humped ♂ femora 5–7, the sternal process present between ♂ coxae 4, occasionally also between ♂ coxae 3, as well as a short gonopod femorite and a strongly condensed solenophore.

#### Description.

**Figure 7.**
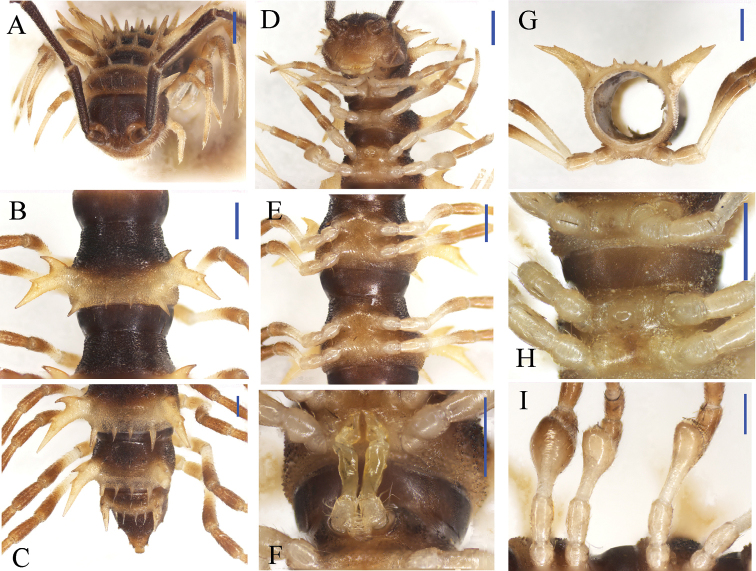
*Desmoxytes nodulosa* sp. n., ♂ paratype from Xia’ao Town, near Xia’ao Middle School, cave II. **A, D** anterior part of body, subdorsal and ventral views, respectively **B, E** midbody segments, dorsal and ventral views, respectively **C** posterior part of body, dorsal view **F** gonopods *in situ*, ventral view **G c** ross-section of a midbody segment, caudal view **H** sternal processes between coxae 3 and 4, ventral view **I** femora 5–7, ventral view. Scale bars: 0.5 mm.

Length ca 19–22 (♂) or 20–23 mm (♀), width of midbody pro- and metaterga together with paraterga 1.0–1.5 and 2.2–2.8 (♂), or 1.8–2.0 and 2.8–3.0 mm (♀), respectively. Holotype 21.0 mm long, 1.5 and 2.5 mm wide on midbody pro- and metaterga, respectively. Coloration of material varying from pallid to rather uniformly dark brownish (Fig. 7A). Head yellowish to dark brownish; antennae and anterior body part often a little darker brownish; paraterga, posterior parts of metaterga, sterna and a few basal podomeres pallid to yellowish (Fig. 7A, D). In width, head > collum > segment 2–4 < 5–18, thereafter body gradually tapering towards telson. Head rather densely setose, epicranial suture distinct (Fig. 7A). Antennae rather long and slender, reaching back until segment 6 (♂) or 5 (♀) when stretched dorsally, antennomeres 5 and 6 each with a compact apicodorsal group of bacilliform sensilla.

**Figure 8.**
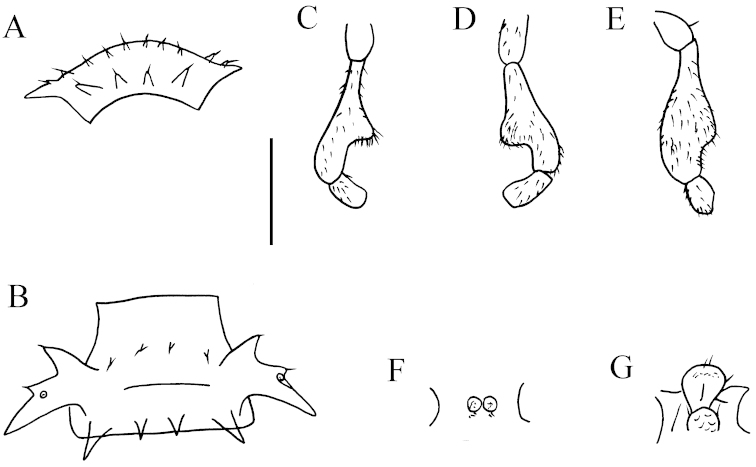
*Desmoxytes nodulosa* sp. n., ♂ paratype from Xia’ao Town, near Xia’ao Middle School, cave II. **A** Collum **B** matatergum 10, dorsal view **C–E** femora 5–7, lateral view **F** sternal processes between coxae 3, ventral view **G** sternal processes between coxae 4, front view. Scale bar: **A–E** = 1.0 mm; **F–G** = 0.5 mm.

Prozonae very delicately microalveolate, but shining; collum, metaterga, paraterga and surface below paraterga finely shagreened and microgranulate, moderately setose in posterior parts of metaterga (Fig. 7B–E). Collum with two transverse rows of coniform spines: 4+4 anterior, 2+2 posterior; paraterga stout and spiniform, directed dorsolaterad, with a spine anteriorly at base (Figs 7A, 8A). Metaterga 2–19 each with 2+2 and 2+2 (or 2+3) coniform spines arranged in two transverse rows, lateral spines of posterior rows much larger than the others in metaterga 2–18 (Figs 7A–C, G, 8B), but of same size on metatergum 19 (Fig. 7C). Paraterga very strongly developed, wing-shaped, usually 3-lobate laterally, occasionally with a setigerous denticle near ozopore, slightly thicker in pore-bearing segments; tip of each paratergal incision with an evident lateral seta (Figs 7B, G, 8B). Paraterga 2–8 directed obliquely upwards at ca 45°, following pareterga growing increasingly horizontal and ending up clearly above dorsum in ♂ (Fig. 7B–C), but slightly lower, shorter, subhorizontal and level to dorsum in ♀. Pore formula normal; ozopores conspicuous, located inside an ovoid groove about 1/3 in front of caudal corner (Figs 7B–C, 8B). Transverse sulcus obscure on collum and metaterga 2–4; more evident, but incomplete on metaterga 5–18 (Figs 7D, 8B). Pleurosternal carinae visible on segments 2 and 3 in both sexes, absent on the rest. Epiproct with 1+1 setigerous knobs on lateral sides, and 2+2 paramedian ones dorsally near midway, tip truncate, lateral pre-apical papillae very distinct, tuberculiform (Fig. 7C). Hypoproct subtrapeziform, caudal margin very slightly concave, setigerous cones at caudal edge very small, widely separated. Axial line missing.

Sterna sparsely setose, cross-impressions visible (Fig. 7E). A rounded subcylindrical sternal process with two small pores between ♂ coxae 4 (Figs 7H, 8G); occasionally a paramedian pair of small, short, rounded tubercles between ♂ coxae 3 as well (Figs 7H, 8F). Legs 1 short, following ones growing increasingly longer and slenderer towards telson, ca 2.2–2.8 (♂) or 2.0–2.2 (♀) times longer than midbody height. ♂ femora 5–7 each with a very strong, rounded, mesal, densely pilose apophysis in distal 1/2 (Figs 7I, 8C–E).

**Figure 9.**
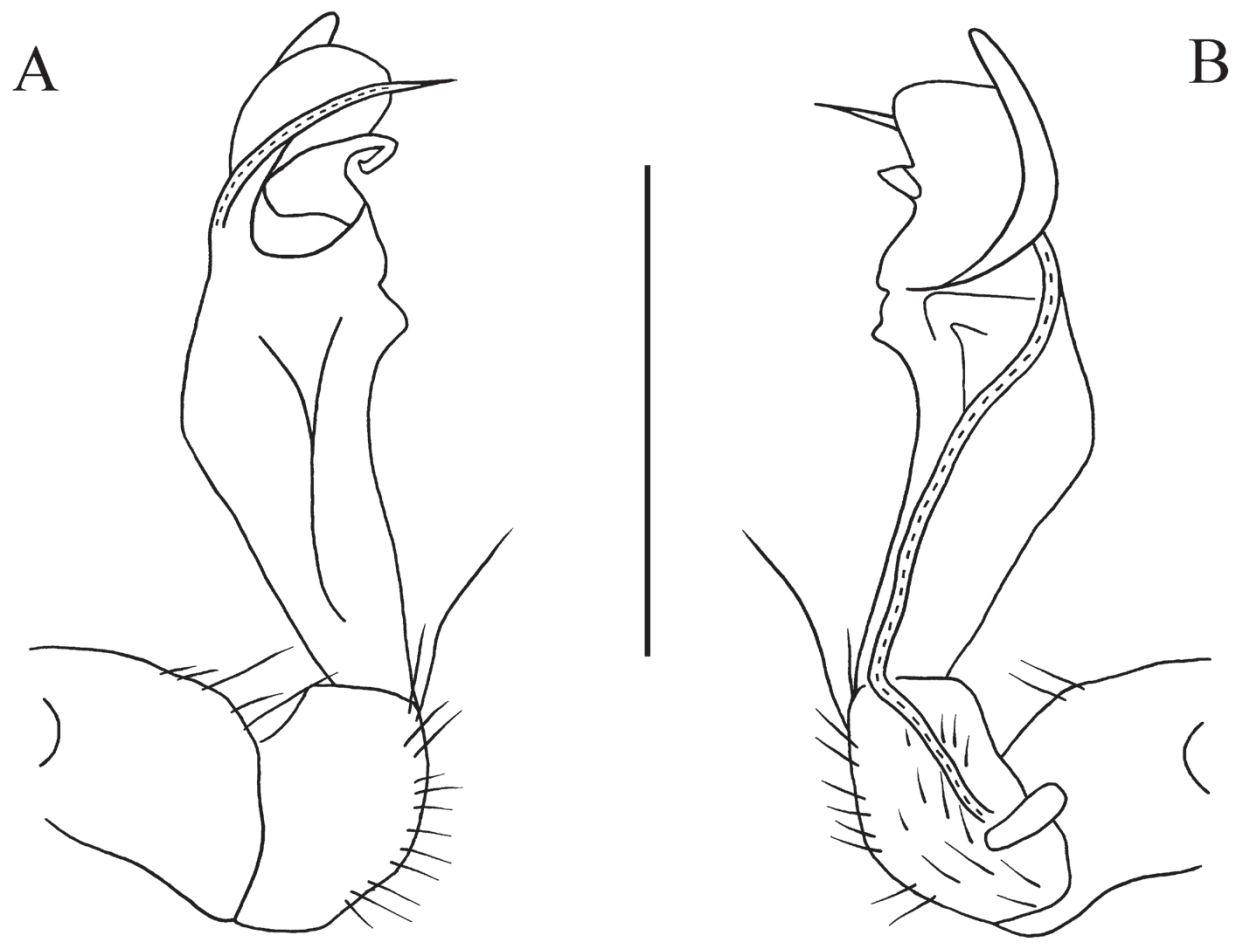
*Desmoxytes nodulosa* sp. n., ♂ paratype from Xia’ao Town, near Xia’ao Middle School, cave II. **A, B** right gonopod, lateral and mesal views, respectively. Scale bar: 0.5 mm.

Gonopods (Figs 7F, 9A, B) short. Coxite short, subcylindrical, poorly setose distodorsally, about 1/3 as long as telopodite. Prefemoral portion less than half as long as acropodite, densely setose. Femorite quite stout, slightly enlarged distad, with seminal groove running entirely on the mesal side, apically with a distinct sulcus demarcating a short, strongly condensed solenophore. Solenomere long, flagelliform, well separated at base from solenophore.

#### Remarks.

Although the coloration of this species is quite variable, based on several troglomorphic traits such as some individuals being completely unpigmented, and the antennae and legs clearly elongated, this species may well be a troglobite.

### Desmoxytes getuhensis sp. n.

Taxon classificationAnimaliaPolydesmidaParadoxosomatidae

http://zoobank.org/D8B16E5A-72FA-4054-9A35-F04EC4EB2FB1

Figs 10 , 11 , 12

#### Holotype.

♂ (SCAU), China, Guizhou Prov., Anshun City, Ziyun County, Getuhe National Geopark, cave Suidao Dong, 25°41.32'N, 106°18.26'E, 950 m, 28.XII.2012, leg. Tian Mingyi, Liu Weixin, Sun Feifei & Yin Haomin.

#### Paratypes.

2 ♂, 5 ♀, 1 ♂ juv., 1 ♀ juv. (SCAU), 1 ♂, 1 ♀ (IZAS), 1 ♂, 1 ♀ (ZMUM), same locality and collectiong data as of the holotype. 1 ♂, 6 ♀ (SCAU), same locality, cave Taiyang Dong, 25°41.55'N, 106°14.27'E, 1056 m, 28.XII.2012, leg. Tian Mingyi, Liu Weixin, Sun Feifei & Yin haomin.

#### Name.

To emphasize the location of the new species within the Getuhe National Geopark.

#### Diagnosis.

Differs from congeners in the paraterga being long and spiniform throughout, and the antennae and legs very long, combined with setose tubercles between ♂ coxae 4, the humped ♂ femur 6, and the gonopods strongly condensed.

#### Description.

**Figure 10.**
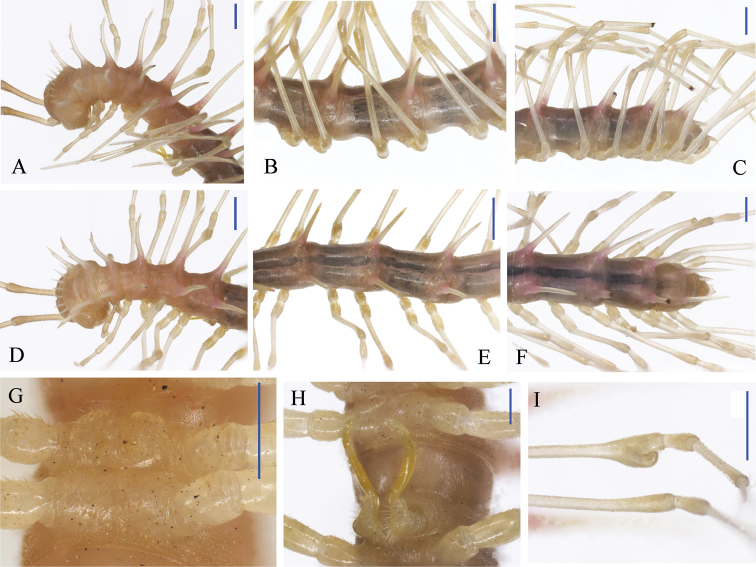
*Desmoxytes getuhensis* sp. n., ♂ paratype from Getuhe National Geopark, cave Taiyang Dong. **A, D** anterior part of body, sublateral and dorsal views, respectively **B, E** midbody segments, lateral and dorsal views, respectively **C, F** posterior part of body, lateral and dorsal views, respectively **G** sternal process between coxae 4 *in situ*, ventral view **H** gonopods *in situ*, ventral view **I** femur 6, lateral view. Scale bars: **A–F, I** = 1.0 mm, **G, H** = 0.5 mm.

Length ca 23–27 (♂) or 25–28 mm (♀); width of pro- and metaterga together with paraterga 1.2–1.4 and 2.5–3.0 (♂) or 1.5–2.0 and 2.8–3.0 mm (♀), respectively. Holotype 26.0 mm long, 1.2 and 2.5 mm wide on midbody pro- and metaterga, respectively. Coloration of material rather uniformly light brownish to nearly pallid, anterior body part a little darker, some specimens pinkish (Fig. 10A–F). Antennomere 7 dark brown. Head broadest, densely setose, but more sparsely so on vertex, epicranial suture distinct (Fig. 10D). Antennae extremely long and slender, reaching back until segment 7 (♂) or 6 (♀) when stretched dorsally, antennomeres 5 and 6 each with a compact apicodorsal group of bacilliform sensilla.

**Figure 11.**
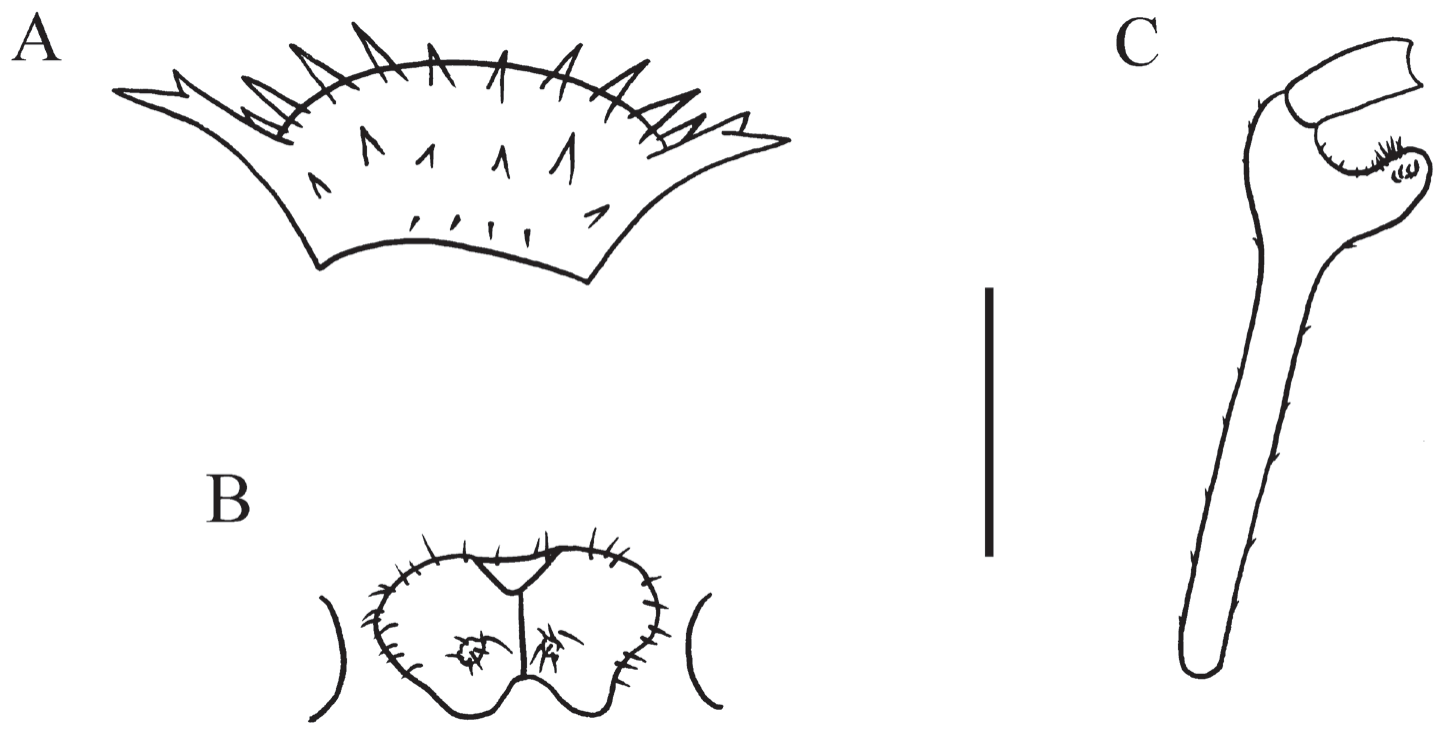
*Desmoxytes getuhensis* sp. n., ♂ paratype from Getuhe National Geopark, cave Taiyang Dong. **A** Collum **B** sternal process between coxae 4, ventral view **C** femur 6, lateral view. Scale bar: **A, C** = 1.0 mm; **B** = 0.5 mm.

Tegument rather shining and smooth, prozonae delicately microalveolate, metaterga and surface below paraterga finely shagreened to microgranulate (Fig. 10A–F). Collum with 5+5 evident spines arranged in a row at front margin, behind it with about 3(2)+3(2) and 2(3)+2(3) smaller spinules in an irregular transverse row; paraterga stout and spiniform, directed dorsolaterad, with a small denticle frontally (Figs 10A, D, 11A). Metaterga 2–4 each with 3+3 and 3+3, similar, but smaller spinules arranged in two transverse rows; sculpture on following metaterga gradually disappearing. Metatergum 19 with 3+3 and 3+3 setae in two rows. Paraterga (Fig. 10A–F) extremely long, straight, spiniform, about as high as body height in ♂, a little shorter in ♀; mainly directed more dorsad than laterad and ending up clearly above dorsum on collum and in segments 2–18; only paraterga 19 subhorizontal, about level to dorsum, directed clearly caudad and reaching behind until about midlength along telson (Fig. 10F). Paraterga 2–4 each with two evident indentations frontally (Fig. 10A, D). Pore formula normal; ozopores inconspicuous, located just at base on lateral side of poriferous paraterga. Transverse sulcus usually very vague, but traceable in segments 5–18 (Fig. 10E–F). Pleurosternal carinae evident only on segments 2 and 3 in both sexes (Fig. 10A), absent on the rest. Epiproct (Fig. 10F) rather simple, lateral pre-apical papillae very distinct, finger-shaped. Hypoproct subtrapeziform, caudal margin emarginate, setigerous cones at caudal edge very large, widely separated. Axial line present.

Sterna quite spasely setose, cross-impressions weak (Fig. 10G–H). A paramedian pair of short, rounded, setose tubercles between ♂ coxae 4 (Figs 10G, 11B). Legs (Fig. 10C) extremely long and slender, ca 3.0–4.0 times longer than midbody height. ♂ femur 6 strongly inflated ventrally in distal 1/5 (Figs 10I, 11C).

**Figure 12.**
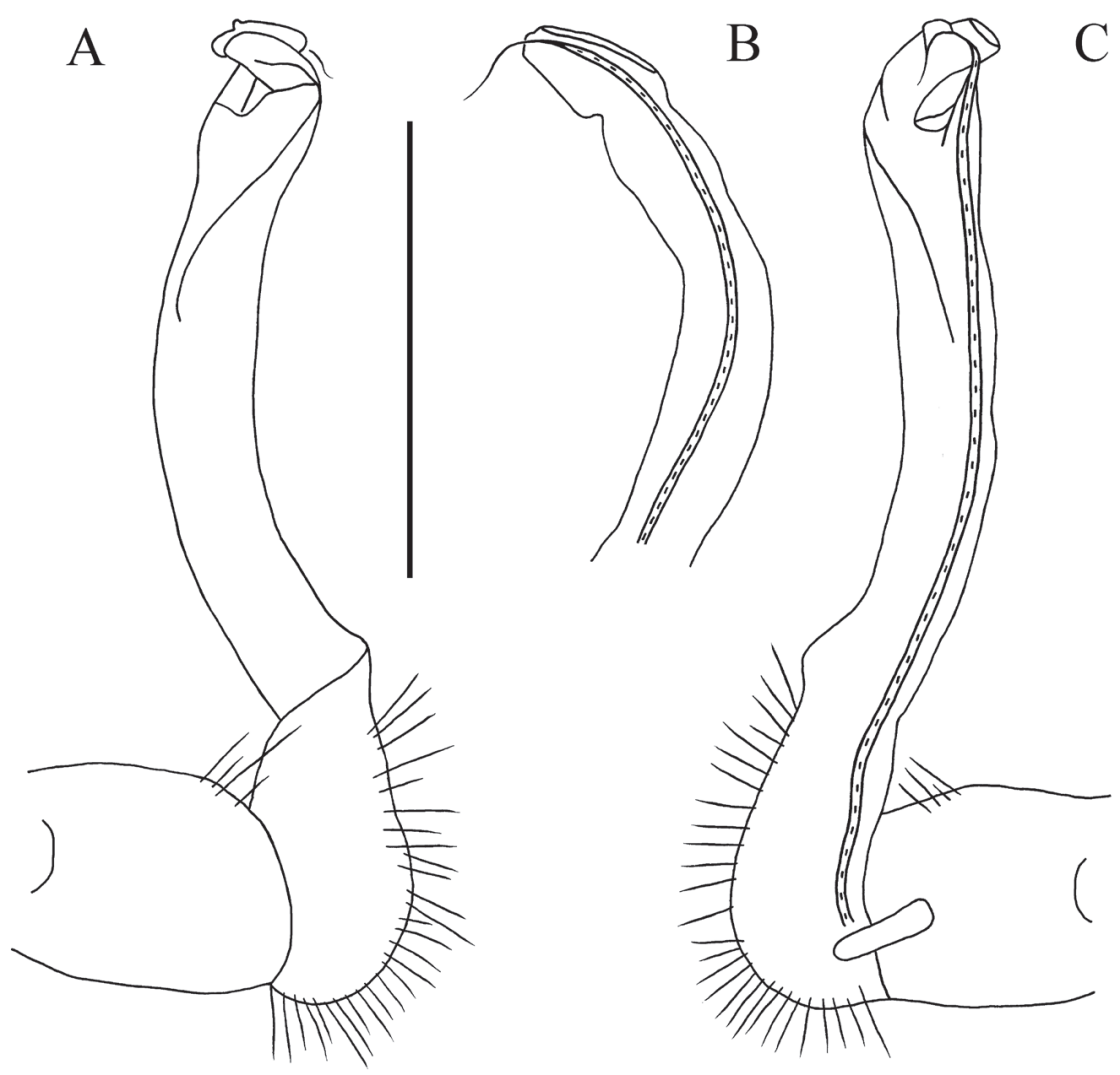
*Desmoxytes getuhensis* sp. n., ♂ paratype from Getuhe National Geopark, cave Taiyang Dong. **A–C** right gonopod telopodite, lateral, dorsal and mesal views, respectively. Scale bar: 0.5 mm.

Gonopods (Figs 10H, 12A–C) simple. Coxite rather short, subcylindrical, poorly setose distodorsally, about 1/3 as long as telopodite. Prefemoral portion about half as long as acropodite, densely setose. Femorite rather slender, elongate, slightly curved, with seminal groove running entirely on the mesal side. Postfemoral part strongly condensed; solenomere short, flagelliform, evidently separated at base from solenophore.

#### Remarks.

Based on several troglomorphic traits such as some individuals being nearly unpigmented, and the antennae and legs very strongly enlongated, this species seems to be a troglobite.

### Desmoxytes scutigeroides

Taxon classificationAnimaliaPolydesmidaParadoxosomatidae

Golovatch, Geoffroy & Mauriès, 2010

Desmoxytes scutigeroides Golovatch, Geoffroy & Mauriès, 2010: 58.
Desmoxytes scutigeroides – Nguyen and Sierwald 2013: 1242.

#### Material examined.

1 ♂, 1 ♀ (SCAU), China, Guangxi, Du’an County, Disu Town, Dading Village, cave II, 23°56.34'N, 108°0.32'E, 26.VI.2013, leg. Tian Mingyi, Lin Wei, Yin Haomin & Huang Sunbin; 1 ♂, 1 ♀, 1 ♀ fragment (SCAU), same county, Longwan Town, Nongqu Village, cave I, 23°56.021 N, 108°10.962 E, 459 m, 27.VI.2013, leg. Tian Mingyi, Liu Weixin, Lin Wei, Yin Haomin & Huang Sunbin.

#### Remarks.

This species has been described from a few caves in Huanjiang County, Guangxi, China while the new samples derive from two caves in the neighbouring Du’an County, Guangxi. The above material is in good agreement with the original description by Golovatch et al. (2010).

### Desmoxytes scolopendroides

Taxon classificationAnimaliaPolydesmidaParadoxosomatidae

Golovatch, Geoffroy & Mauriès, 2010

Desmoxytes scolopendroides Golovatch, Geoffroy & Mauriès, 2010: 60.
Desmoxytes scolopendroides – Nguyen and Sierwald 2013: 1242.

#### Material examined.

1 ♀ (SCAU), China, Guangxi, Du’an County, Gaoling Town, Jinzhu Village, cave I, 24°06.547'N, 108°04.785'E, 190 m, 3.V.2013, leg. Tian Mingyi; 1 ♀ (SCAU), same locality, cave II, 24°06.514'N, 108°04.695'E, 218 m, 3.V.2013, leg. Liu Weixin; 5 ♂, 5 ♀ (SCAU), same county, Xia’ao Town, cave I, 24°15.144'N, 107°56.272'E, 347 m, 2.V.2013, leg. Tian Mingyi, Liu Weixin, Sun Feifei & Yin Haomin; 1 ♀, 4 ♂ juv., 8 ♀ juv. (SCAU), same cave, 28.VI.2013, leg. Tian Mingyi, Liu Weixin, Lin Wei, Yin Haomin & Huang Sunbin; 3 ♂, 3 ♀ (SCAU), same cave, 28.XII.2013, leg. Tian Mingyi, Liu Weixin, Yin Haomin & Luo Xiaozhu.

#### Remarks.

This species has been described from a cave in Huanjiang County, Guangxi, China while the new samples come from a few more caves in the neighbouring Du’an County, Guangxi. The above material is in good agreement with the original description by Golovatch et al. (2010).

### A key to *Desmoxytes* species in China

**Table:** 

1	Paraterga spiniform, mostly very long and directed evidently more dorsad than laterad (Figs 4A–D, 10A–F)	**2**
–	Paraterga wing- (Fig. 7A–C) or antler-shaped (Fig. 1D–F)	**8**
2	Adult body relatively small, length <20 mm	***Desmoxytes parvula* sp. n.**
–	Adult body much larger, length >20 mm	**3**
3	Paraterga long and spiniform only on collum and following four segments, evidently shorter on segment 5, small and coni- to tuberculiform thereafter	***Desmoxytes lui***
–	Paraterga subequally long and spiniform at least in segments 2–18 (Fig. 10A–F)	**4**
4	Only ♂ femur 7 very evidently humped distoventrally	***Desmoxytes longispina***
–	Either ♂ femur 6 or both femora 6 and 7 very evidently humped distoventrally	**5**
5	Both ♂ femora 6 and 7 very evidently humped ventrally in distal quarter	***Desmoxytes spinissima***
–	Only ♂ femur 6 very evidently humped distoventrally	**6**
6	Metaterga not only with normally arranged setigerous tubercles, but also with a row of similar tubercles along posterior rim	***Desmoxytes minutubercula***
–	Metaterga 2–4 with several transverse rows of setigerous spines, following metaterga smooth, sculpture gradually disappearing	**7**
7	Gonopods telopodite subfalcate, femorite stouter relative to a condensed solenophore; Guangxi	***Desmoxytes scutigeroides***
–	Gonopods (Figs 10H, 12A–C) more simple, only slightly curved, femorite rather slender and elongate relative to a particularly short solenophore; Guizhou	***Desmoxytes getuhensis* sp. n.**
8	Paraterga wing-shaped	**9**
–	Paraterga antler-shaped	**12**
9	♂ femora unmodified	***Desmoxytes eupterygota***
–	At least a pair of ♂ femora (5–7) humped	**10**
10	Metaterga 2–19 with only two transverse rows of 2+2(3) setigerous spines	**11**
–	Metaterga 2–19 with more than two transverse rows of setigerous spines	***Desmoxytes scolopendroides***
11	♂ femora 5–7 very evidently humped distoventrally (Figs 7I, 8C–E); a single sternal process between ♂ coxae 4 (Figs 7H, 8G)	***Desmoxytes nodulosa* sp. n.**
–	♂ femora 5 and 6 slightly humped distoventrally; two processes between ♂ coxae 4	***Desmoxytes planata***
12	Paraterga 2–18 antler-shaped, evidently branched; Jiangxi	***Desmoxytes draco***
–	Anterior paraterga evidently antler-shaped, posterior paraterga rather long and spiniform, evidently 2- or 3-dentate laterally; Guangxi	**13**
13	A pair of setose tubercles between ♂ coxae 3, and a peculiar linguiform sternal process between ♂ coxae 5 (Figs 1G, 2E)	***Desmoxytes lingulata* sp. n.**
–	A pair of bristle-like tubercles between ♂ coxae 3, and a very deeply divergent sternal process between ♂ coxae 5	***Desmoxytes cornuta***

## Supplementary Material

XML Treatment for Desmoxytes lingulata

XML Treatment for Desmoxytes parvula

XML Treatment for Desmoxytes nodulosa

XML Treatment for Desmoxytes getuhensis

XML Treatment for Desmoxytes scutigeroides

XML Treatment for Desmoxytes scolopendroides
